# Discrepancies among equations to estimate the glomerular filtration rate for drug dosing decision making in aged patients: a cross sectional study

**DOI:** 10.1007/s11096-023-01677-8

**Published:** 2023-12-27

**Authors:** M. Margarida Castel-Branco, Marta Lavrador, Ana C. Cabral, Adriana Pinheiro, Joana Fernandes, Isabel Vitória Figueiredo, Fernando Fernandez-Llimos

**Affiliations:** 1 Pharmacology and Pharmaceutical Care Laboratory, Faculty of Pharmacy, University of Coimbra, Coimbra, Portugal; 2 Institute for Clinical and Biomedical Research (iCBR), Faculty of Medicine, University of Coimbra, Coimbra, Portugal; 3 USF Serra da Lousã, Lousã, Portugal; 4 Laboratory of Pharmacology, Department of Drug Sciences, Faculty of Pharmacy, University of Porto, Porto, Portugal; 5 Applied Molecular Biosciences (UCIBIO), University of Porto, Porto, Portugal

**Keywords:** Aged, Drug dosage calculations, Glomerular filtration rate, Patient safety, Renal Insufficiency

## Abstract

**Background:**

Patients often require adjustments to drug doses due to impaired renal function. Glomerular filtration rate (GFR) estimation using various equations can result in discrepancies, potentially leading to different dose adjustment recommendations.

**Aim:**

To determine the clinical significance of discrepancies observed between different equations used to estimate GFR for drug dose adjustments in a real-world group of patients over 65 years in primary care.

**Method:**

The Cockcroft–Gault (CG), Modification of Diet in Renal Disease (MDRD), Chronic Kidney Disease Epidemiology Collaboration (CKD-EPI), and Berlin Initiative Study 1 equations were applied to estimate GFR in a group of patients over 65 years old attending a primary care center. Results were compared using Bland–Altman plots, and limits of agreement (LoA) and overall bias were calculated. Regression analyses were conducted to identify the null difference GFR and the slope of differences for each pairwise comparison.

**Results:**

A total of 1886 patients were analyzed. Differences between patient-adjusted and body surface area (BSA)-normalized versions of the equations were not clinically relevant for dose adjustments, with LoAs below 20 mL/min. However, discrepancies among the original versions of several equations presented LoAs over 30 mL/min. Greater differences were found between CG and MDRD or CKD-EPI equations.

**Conclusion:**

Clinically relevant differences in GFR estimation were observed among different equations, potentially impacting drug dose adjustments. However, discrepancies were not considered significant when comparing patient-adjusted and BSA-normalized versions of the equations, particularly for patients with BSA close to the average.

**Supplementary Information:**

The online version contains supplementary material available at 10.1007/s11096-023-01677-8.

## Impact statements

- Patient-adjusted and body surface area (BSA)-normalized versions of the equations can be used interchangeably in patients with an average BSA.
- The use of different equations to estimate glomerular filtration rate may result in discrepant recommendations for drug dose adjustments.

## Introduction

The rise in life expectancy, with the subsequent increase in patients with multimorbidity, has led to an increase in polypharmacy, thereby elevating the risk of inappropriate prescriptions within the aged population [1, 2]. This population usually experiences impaired renal function, which directly affects the elimination of drugs through the kidneys. From a patient safety perspective, accurately estimating renal function in medication users is crucial to minimize the occurrence of potentially inappropriate prescription of renally excreted drugs in clinical practice [3, 4].

Glomerular filtration rate (GFR) is widely recognized as the most reliable indicator of kidney function. A persistent decline in GFR below 60 mL/min/1.73 m^2^ indicates the presence of chronic kidney disease, while a consistent GFR between 60 and 90 mL/min/1.73 m^2^ warrants further investigation [5]. Exogenous filtration markers like inulin, iohexol, or iothalamate offer the most accurate means of measuring GFR, but their practicality in clinical settings is limited. Creatinine clearance (CrCl), calculated by measuring the concentration of creatinine in a 24-h urine collection and the serum creatinine (SCr) level, can serve as an alternative for estimating GFR [6–8]. However, it is important to note that CrCl as a proxy for GFR can be influenced by factors such as the patient's muscle mass, nutritional status, physical activity, or liver conditions.

In clinical practice, CrCl and GFR are typically estimated rather than directly measured. Various equations have been developed that incorporate patient-specific variables such as SCr, body weight, race, age, and sex to estimate renal function at a steady-state condition. The Cockcroft–Gault (CG) equation, widely used in drug development [8], was initially validated using CrCl measured in a 24-h urine collection as the gold standard [6]. The Modification of Diet in Renal Disease (MDRD) equation [9] and the Chronic Kidney Disease Epidemiology Collaboration (CKD-EPI) equation [10, 11] have been commonly used to estimate GFR (eGFR) since they were validated using iothalamate as the gold standard. Additionally, the Berlin Initiative Study 1 (BIS1) equation was validated with iohexol to estimate GFR specifically in elderly individuals above 70 years old [12]. There is no universally preferred equation for estimating renal function, and all these formulas are considered acceptable in individuals with relatively stable renal function, despite yielding different results [8, 13–17].

Regardless of the equation used, differences with measured GFR will always exist due to different reasons. Equations using creatinine are less accurate when kidney function reduces to very low values, because creatinine is filtered but also actively secreted by tubular cells in the kidneys (through transporters such as OAT [18], OCT [19], MATE1 and MATE2K [20]). This should be considered when using creatinine-based equations for drug dosing in severe impairment [21].

The utilization of different equations for estimating GFR can lead to discrepant drug dose adjustments [8, 17, 22–24]. Furthermore, the instructions provided in the Summaries of Product Characteristics (SmPCs) approved by the European Medicines Agency or the US Food and Drug Administration for dose adjustment in patients with renal impairment do not specify the equation used to estimate GFR in their recommendations [8, 25]. The clinical significance of the variability in GFR estimates requires further investigation [17, 23, 26].

### Aim

The aim of this study was to determine the clinical significance of discrepancies observed among different equations used to estimate GFR for drug dose adjustments of renally excreted drugs in a real-world group of patients over 65 years of age in primary care.

### Ethics approval

The study was conducted in compliance with the principles outlined in the Declaration of Helsinki and received approval from the Ethics Committee of the Center Portugal Regional Health Administration (registration number: CE-19/2022). In accordance with Portuguese legislation, informed consent for the use of secondary data from medical records is not required if the study is approved by an ethics committee and if the data are appropriately anonymized.

## Method

### Study design

This cross-sectional study analyzed the entire population over 65 years of age attending a Family Care Center (Unidade de Saúde Familiar—USF) in the Central Region of Portugal. Data were extracted from the USF medical records on February 1st, 2022. The extracted data included patients' demographic information (age, gender), physical parameters (body mass, height), prescribed medications (including dates), diagnosed medical conditions (including date of diagnosis), and the results of the most recent SCr test (including the date of the test). The data extraction process was carried out by a USF physician who had full access to the patients' medical records. To ensure anonymity, an unambiguous record number was assigned to each patient in the extracted file. As a result, individuals were no longer identifiable in the database, except for the extracting physician who retained the deanonymization key to re-identify a patient if any relevant information pertaining to their health emerged during the research.

The study included individuals aged 65 years and above who were taking at least one medication and had visited the USF within the last 2 years. To determine the date of the last visit, a composite variable was created by selecting the most recent date among the following: the date of the last prescribed medicine, the date of the last serum creatinine exam, and the date of the last recorded medical condition.

### Instruments

Body mass index (BMI) through the Quetelet index [27], body surface area (BSA) with the Du Bois equation [28], and ideal body weight (IBW) with Friesen equation [29] were calculated using the following equations:

$$ \begin{aligned} & BMI = \frac{{weight_{in\_Kg} }}{{\left( {stature_{in\_m} } \right)^{2} }} \\ & BSA = weight_{in\_Kg}^{0.425} *stature_{in\_cm}^{0.725} *0.007184 \\ & IBW = \frac{weight*22}{{BMI}} \\ \end{aligned} $$

Five equations were employed to estimate GFR in the study: CG using actual body weight, CG using IBW, MDRD, CKD-EPI 2021 version, and BIS1. The estimated GFR (eGFR) equations used were:

$$ CG = \frac{{\left( {140 - age} \right)* weight_{{in_{Kg} }} }}{72*SCr}*A $$

A = 0.85 if female

$$ MDRD = 175*SCr^{ - 1.154} *age^{ - 0.203} *A*B $$

A = 1.212 if black; B = 0.742 if female

$$ CKD EPI = 141*\min \left( {\frac{SCr}{K},1} \right)^{\alpha } *\max \left( {\frac{SCr}{K},1} \right)^{ - 1.209} *0.933^{age} *A $$

If female: K = 0.7; α = -0.329; A = 0.018.

If male: K = 0.9; α = -0.411; A = 1.159

$$ BIS1 = 0.3736*SCr^{ - 0.87} *age^{ - 0.95} *A $$

A = 0.82 if female.

The two CG equations are adjusted based on the patient's body weight, and the results are expressed in mL/min. In contrast, the remaining equations are normalized to BSA and expressed in mL/min/1.73 m^2^. To facilitate comparison between these different types of equations, as described by Sharma et al. [8] and Khanal et al. [17], an adjusted version of the normalized equations was calculated. This adjustment involved multiplying the normalized result by each patient's BSA and dividing it by 1.73. Conversely, an inverse calculation was performed to obtain a normalized version of the two CG equations.

### Data analysis

A descriptive analysis was performed, presenting absolute and relative values for categorical variables, as well as means and standard deviations (SD) or medians and interquartile ranges (IQR) for continuous variables, depending on their normality. Normality was assessed using the Shapiro–Wilk test, in conjunction with a visual inspection of the quantile–quantile (Q–Q) plot. Calculations were carried out using SPSS v.28 (IBM, Armonk, NY, USA). No imputation of missing data was performed.

To assess the agreement between two different equations, the visual analysis method proposed by Bland and Altman was employed [30, 31]. The limits of agreement were defined as ± 1.96 times the standard deviation (SD), indicating that 95% of the distribution falls within these limits. The bias was calculated as the average of the differences between the two methods. The percentage of error was determined by calculating the distance between the two limits of agreement relative to the mean value of the first method being compared. To complete the Bland–Altman plot, a linear regression analysis was performed using the discrepancies (represented by the differences) as the dependent variable and the average eGFR value as the independent variable. The slope of the regression represents the scale dimension and the direction of proportionality between the error and the eGFR. Additionally, this regression analysis facilitated the determination of the average eGFR value where the discrepancy between the equations equals zero. This value corresponds to the eGFR point at which one equation transitions from underestimating to overestimating the other.

The analysis initially involved pairwise comparisons between the adjusted and normalized versions of the five equations. Subsequently, similar analyses were repeated to compare the original versions of the five equations: CG(a), CG-IBW(a), MDRD(n), CKD-EPI(n), and BIS1(n), in pairwise comparisons.

In order to examine the impact of patients' physical characteristics on the discrepancies between patient-adjusted and BSA-normalized equations, a linear regression analysis was conducted for each paired comparison. The difference between the estimates from the two equations was used as the dependent variable, while BMI and BSA served as the independent variables. Bland–Altman plots and bias calculations were performed using R/RStudio (Posit, Boston, MA) with the assistance of the ggplot2 package (https://cran.r-project.org/package=ggplot2). The creation of linear regression models was carried out in Excel (Microsoft, Redmond, WA, USA) using the Analysis ToolPak add-in.

## Results

The original database obtained from the computerized medical record system of the USF consisted of 3061 patients who had at least one medication prescribed. Out of these, 1175 patients were excluded as they had not visited the USF in the 2 years preceding the study. Ultimately, a total of 1886 patients were included for analysis, and their characteristics are presented in Table 1.

**Table 1:** Characteristics of included patients

	Number valid data	Mean (SD) or Median [IQR]^a^
Gender (female)	1886	1077 (57.1%)
Age (years)	1886	76.6 (7.9)
Num. medicines prescribed	1886	6.4 (4.5)
Days from last visit	1886	108 [50: 206]
Serum creatinine	1860	0.8 [0.8: 1.1]
Days from creatinine exam	1860	631 [510: 804]
Body weight (Kg)	1842	73.9 (14.1)
Stature (cm)	1839	162 (8.5)
Body mass index (Kg/m^2^)	1839	28.1 (4.8)
Body surface area (m^2^)	1839	1.78 (0.19)
Ideal body weight (Kg)	1839	58.0 (6.1)
Weight over IBW (Kg)	1839	15.9 (12.5)
Cockcroft–Gault eGFR (mL/min)	1838	67.9 (23.9)
Cockcroft–Gault w/IBW eGFR (mL/min)	1835	53.0 (16.6)
MDRD eGFR (mL/min/1.73 m^2^)	1860	71.7 (20.5)
CKD-EPI eGFR (mL/min/1.73 m^2^)	1860	73.9 (18.7)
BIS1 eGFR (mL/min/1.73 m^2^)	1860	61.2 (15.5)

*IBW* ideal body weight, *eGFR* estimated glomerular filtration rate, *MDRD* modification of diet in renal disease, *CKD-EPI* chronic kidney disease epidemiology collaboration, *BIS1* Berlin Initiative Study 1

^a^*SD* standard deviation, *IQR* interquartile range (presented according to normality of data)

When comparing the patient-adjusted and BSA-normalized versions of the equations (Table 2), a positive but small bias was observed for each paired comparison. The bias ranged from 1.49 mL/min when using IBW in the CG equation to 2.52 mL/min when using actual body weight, indicating a moderate overall underestimation of GFR when employing BSA-normalized equations. The limits of agreement were also moderate, with all comparisons falling within the range of ± 20 mL/min. The regression slopes were all positive and small. In all equations, the regression line intersected the null difference in average GFR at clinically relevant values for dose adjustments, ranging from 30 mL/min in CG with IBW to 58 mL/min in the CKD-EPI equation. Bland–Altman plots can be found in Supplementary File 1.

**Table 2:** Bland–Altman analysis of agreement between patient-adjusted and body surface area-normalized equations to estimate glomerular filtration rate

	Bias	Error (%)	Limit of agreement		Regression		x when y = 0	
			Upper	Lower	R square	Equation	x	95% CI
CG(a) and CG(n)	2.52	43.83	17.41	− 12.37	0.222	y = 0.16x − 8.39	51	[42: 63]
CG_IBW(a) and CG_IBW(n)	1.49	42.29	12.70	− 9.72	0.034	y = 0.07x − 1.99	30	[13: 57]
MDRD(a) and MDRD(n)	2.21	43.08	18.12	− 13.69	0.074	y = 0.10x − 5.33	51	[34: 76]
CKD-EPI(a) and CKD-EPI(n)	2.34	42.53	18.55	− 13.87	0.100	y = 0.13x + 7.70	58	[41: 79]
BIS1(a) and BIS1(n)	1.94	42.56	15.38	− 11.50	0.095	y = 0.13x − 6.14	47	[33: 66]

*CG* Cockcroft–Gault, *CG-IBW* Cockcroft–Gault with ideal body weight, *MDRD* Modification of diet in renal disease, *CKD-EPI* chronic kidney disease epidemiology collaboration, *BIS1* Berlin initiative study 1, *BSA* body surface area

(a) Patient-adjusted equations; (b) BSA-normalized equations

**Fig. 1:**
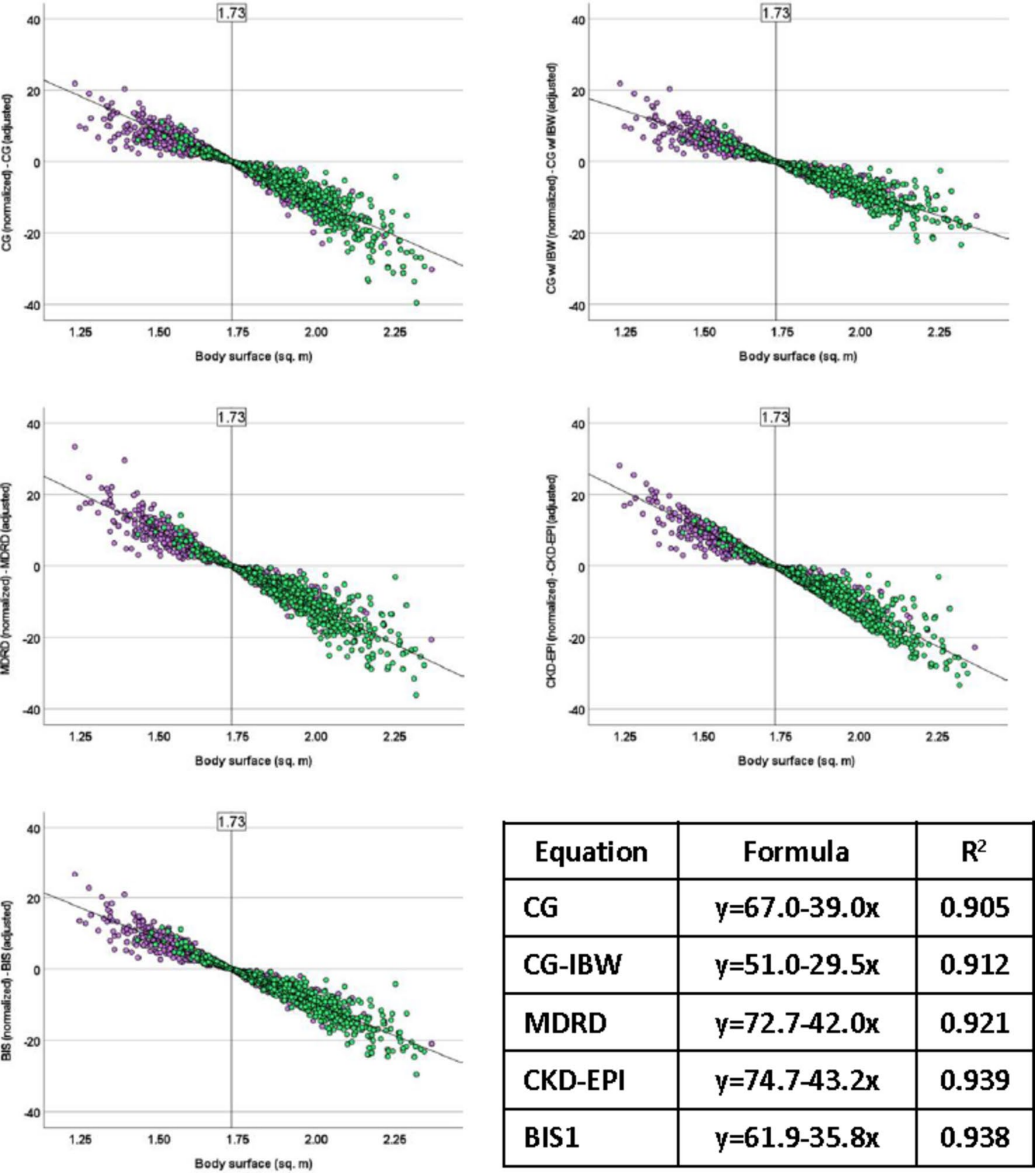
Regression analysis of distance between patient-adjusted and body surface area-normalized estimate glomerular filtration rate with body surface area as independent variable. Male: green dots, purple dots: female. *CG* Cockcroft–Gault, *CG-IBW* Cockcroft–Gault with ideal body weight, *MDRD* modification of diet in renal disease, *CKD-EPI* chronic kidney disease epidemiology collaboration, *BIS1* Berlin Initiative Study 1

**Fig. 2:**
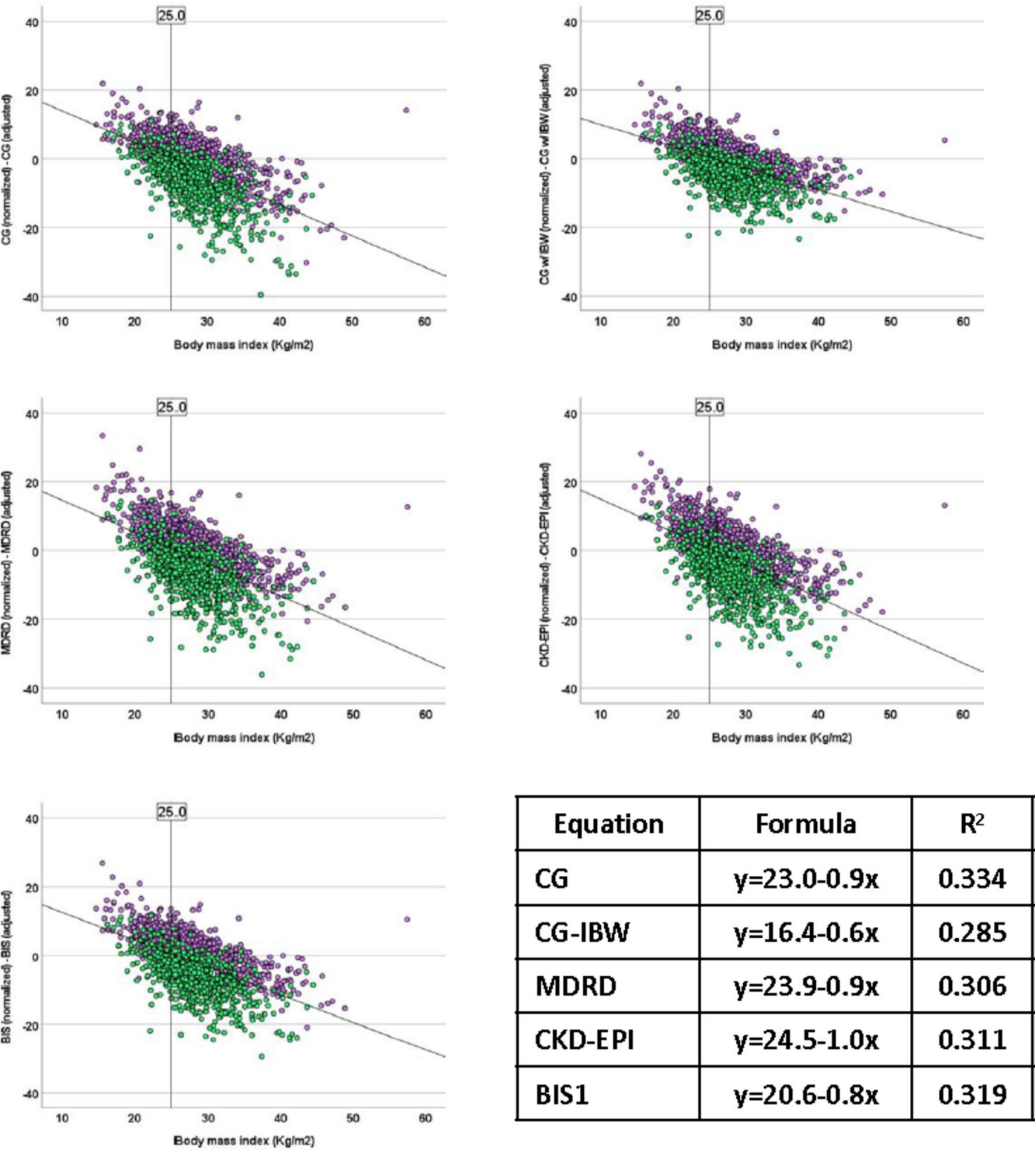
Regression analysis of distance between patient-adjusted and body surface area-normalized estimate glomerular filtration rate with body mass index as independent variable. Male: green dots, purple dots: female. *CG* Cockcroft–Gault, *CG-IBW* Cockcroft–Gault with ideal body weight, *MDRD* modification of diet in renal disease, *CKD-EPI* chronic kidney disease epidemiology collaboration, *BIS1* Berlin Initiative Study 1

The regression analyses of the differences between the two versions of the five equations with BSA as an independent variable demonstrated strong adjustments, with R^2^ values consistently exceeding 0.9 (Fig. 1). The slopes of the regression lines were moderate. Utilizing the obtained regression equations, the BSA confidence intervals necessary to achieve a maximum variability of ± 10 mL/min were as follows: CG 1.46–1.97; CG-IBW 1.39–2.07; MDRD 1.49–1.97; CKD-EPI 1.50–1.96; and BIS1 1.45–2.01. Conversely, the regression analyses of the five paired comparisons with BMI as an independent variable (Fig. 2) yielded much lower R^2^ values, approximately around 0.3.

The analysis of agreement between pairs of the original versions of the equations revealed significant variability (Supplementary File 2). The highest bias was observed in the comparison between CG_IBW and CKD-EPI, while the lowest bias was found in the comparison between MDRD and CKD-EPI. The widest limits of agreement were observed in the comparison between CG and CG_IBW. The comparison between CG and MDRD exhibited the highest percentage of error, and the comparison between CG and BIS demonstrated the steepest regression slope. Notably, the comparisons between CG and BIS1, CG and CG_IBW, and MDRD and BIS1 displayed regression lines intersecting the x-axis (y = 0) within a clinically relevant range for dose adjustments.

## Discussion

### Statement of key findings

The comparison between patient-adjusted and BSA-normalized versions of the equations demonstrated a small underestimation of GFR by the BSA-normalized versions. However, the limits of agreement, calculated as 1.96 times the standard deviation, consistently remained below 20 mL/min. This indicates that the differences between the two versions of the equations were within the recommended 20% precision limit [32, 33].

In contrast, when comparing the results of the five original equations, notable differences were observed, with limits of agreement close to ± 40 mL/min. This suggests that the selection between a normalized or adjusted version of an eGFR equation may be of less significance for dose adjustments compared to the choice of one of the five equations themselves.

### Strengths and weaknesses

We conducted a comparative analysis of the different equations used to estimate GFR using the Bland–Altman plot, which is recommended for such comparisons. In addition to the visual analysis of the plot, we calculated additional statistics to assess the distribution of differences between the paired instruments. These statistics helped determine the limits of agreement, which represent the average discrepancy between the two instruments across the entire range of measured values in the analyzed patients. Furthermore, we calculated the regression line of the difference distribution, which precisely identifies the GFR value at which the measurements obtained with the two instruments diverge in magnitude. This information is crucial in assessing the clinical relevance of the discrepancy and its potential impact on dose adjustment procedures. Moreover, by employing regression analyses of the discrepancies with anthropometric measures, we were able to establish the range of BSA where adjusted and normalized equations can be interchangeably used.

A key strength of our study was the utilization of a real-world group of patients comprising aged individuals attending a primary care center in central Portugal. This approach allowed us to evaluate the practical relevance of the different equations in a real-life setting bearing in mind the typical limitations of a primary care setting with routine care procedures. In this environment, clinicians must make decisions in a limited amount of time, with limited resources available, where clear recommendations contribute to enhanced patient safety. Additionally, studies reported that using clinical decision support systems (CDSS) in drugs with definite dose recommendations produce better results than in drugs without definite dose recommendations [34]. However, it is important to note that these patients may not be fully representative of all aged patients globally or even within Portugal. One characteristic of studies that employ secondary data obtained from medical records is the reliance on healthcare providers outside the research team for data reliability. Furthermore, it is important to acknowledge that some data was missing, which is a common challenge in studies of this nature. It is essential to clarify that our study did not aim to identify the best possible equation, as this was not within the scope of our research. Additionally, we did not validate the results against a gold standard method for measuring GFR.

Another strength of the study is the use of simple formulas to fit in primary care setting needs. Quetelet and Du Bois are the accepted gold standard equations to estimate BMI and BSA, respectively. In the absence of an accepted gold standard to calculate IBW [35], we preferred using Friesen’s formula, instead of Devine’s [36], because they are equivalent for practical purposes [29] but much easier to use [37].

### Interpretation and further research

Regulatory agencies recommend adjusting drug doses based on absolute (patient-adjusted) GFR rather than BSA-normalized GFR [38]. This recommendation is grounded on the understanding that renal drug clearance is proportional to an individual's GFR [8, 17]. However, for the diagnosis and staging of renal disease, BSA-normalized GFRs are commonly used to categorize the degree of renal impairment [5]. It is worth noting that computer systems often display calculators that provide both patient-adjusted and BSA-normalized estimated GFR equations. This flexibility is intended to accommodate situations where weight and height data are not available [23]. Our study revealed that the discrepancies between patient-adjusted and BSA-normalized equations for estimating GFR are not highly significant. The limits of agreement, which represent the overall discrepancies across the entire range of measurements, were below the commonly accepted ± 20% precision limits considered acceptable [32, 33]. Importantly, the differences between each adjusted-normalized pair were smaller in the clinically relevant range of the GFR interval (below 60 mL/min) than in areas where dose adjustments are not expected (Supplementary File 1) [5].

Normalizing GFR calculations may be valuable for comparative population studies, but it may not be as relevant for dose adjustment practices. This is because higher body weight does not necessarily correspond linearly to greater muscle mass, kidney volume, or the number of functioning nephrons [8]. The literature suggests that considering the difference between the two equations becomes more important when patients deviate significantly from the average body size (BSA around 1.73 m^2^) [17, 23, 39]. To establish the precise limits where patient-adjusted and BSA-normalized eGFR can be interchangeably used, we conducted two regression analyses of the discrepancies between the two equations using two simple anthropometric measures: BSA and BMI. While BMI is commonly used in clinical practice, BSA exhibited a stronger and more reliable association with discrepancies between the instruments. Consequently, using BSA allowed us to identify the specific limits where differences between the two equations remained below recommended ± 10 mL/min [40], that varied from 1.39 to 1.50 m^2^ (lower limit) and from 1.96 to 2.07 m^2^ (upper limit) depending of the equation used (Fig. 1).

The differences observed in eGFR values obtained using the original versions of the equations were greater than those between the adjusted and normalized versions of each equation. In the overall comparisons across the entire interval, the CG equation exhibited the largest discrepancies, as indicated by wider limits of agreement, with MDRD, CKD-EPI, and BIS1 (limits ranging from 25 to 35 mL/min). These differences could be clinically relevant for dose adjustment considerations, especially when the eGFR approaches the nearest cutoff value [23]. While CG was the first equation considered in official prescribing information, there is no consensus on which equation to prefer when discordance is observed [17, 41]. On the other hand, as previously reported, the MDRD and CKD-EPI equations produced similar eGFR results [42, 43]. However, some of these comparisons present an additional challenge that complicates simplistic corrections based solely on overall bias. For instance, the overall bias of the comparison between MDRD and BIS1 is 10.4, suggesting that, on average, MDRD overestimates eGFR by approximately 10 mL/min (Supplementary File 2). However, the regression line intercepts the null difference at 30 mL/min (CI 95% 25:35), indicating that below that value, MDRD underestimates eGFR when compared to BIS1. It is important to pay special attention when the regression line of the discrepancies intercepts the null difference in an area where dose adjustments are expected. Many drugs have dose adjustment recommendations that can lead to different dosing regimens based on different eGFR equations, resulting in shifting estimations between 30 and 60 mL/min. For example, European SmPCs recommend that the normal dose of 2000 mg of metformin should be reduced to 1000 mg when GFR is less than 45 mL/min, or the recommended dose of 5 mg of apixaban is reduced to 2.5 mg when GFR is less than 30 mL/min, and the normal dose of rosuvastatin (10–20 mg) should be reduce to 5 mg if GFR < 60 mL/min. Literature reported than differences in eGFR calculated using the MDRD and BIS1 equations could lead to doubling the dose depending on the equation used [17, 23].

Considering the wide variation in eGFR values among the different equations, our study does not provide sufficient evidence to advocate for the use of one SCr-based equation over another. Instead, a comprehensive evaluation of clinical outcomes resulting from discordant dose recommendations would be necessary to gain further insights and make informed decisions [44]. Alternatively, the use of serum cystatin C–based equations (e.g., CKD-EPI cystatin C [45]) or the combined equations (e.g., CKD-EPI Scr-Scys combined formula [46]) could improve the eGFR calculations, especially at early stages of kidney dysfunction [47]. However, these equations are less likely to be useful in primary care where Serum cystatin C is not routinely measured.

Literature demonstrated that different equations were more accurate than others in different situations. For example, MDRD is preferred to CKD-EPI in low eGFR [8]. Or MDRD and CKD-EPI have a better performance in obese patients than CG [48]. Also, MDRD and CKD-EPI without any race coefficient performed well in sub-Saharan black populations [49]. But considering a primary care setting and aiming to estimate GFR to adjust the dose of renally excreted drugs, using different equations according to the differential characteristics of each patient may not be appropriate. CG was created using isotope dilution mass spectrometry (IDMS)-nontraceable creatinine data and should not be used with IDMS-traceable SCr tests, where results may vary between 10 and 20% [50]. Additionally, CG is the most affected equation in low eGFR due to weight of the creatinine tubular secretion. Subsequently, KDIGO recommends against CG equation for dose adjustments [13]. BIS1 was created and validated to be used in patients over 70 years of age, which limits its generalizability.

MDRD was originally validated using IDMS-nontraceable SCr tests, but then was adapted to IDMS-traceable SCr tests by modifying the coefficients. CKD-EPI was created to be used with IDMS-traceable SCr tests. Thus, both equations may underestimate GFRs if used with IDMS-nontraceable SCr tests. Between these two equations, CKD-EPI is more accurate than MDRD in GFR > 60 mL/min [10], with official bodies suggesting never reporting eGFR > 60 with MDRD [51]. Below 60 mL/min, estimates obtained with MDRD could be slightly more accurate than CKD-EPI, but discrepancies were reported to be negligible [10]. Our results confirm this pattern, with discrepancies below 10 mL/min in the lowest part of the interval, while substantial discrepancies in high GFR (Supplementary File 2). It seems that, if we want to use only one equation in primary care, coincidently with KDIGO, the CKD-EPI could be the best choice.

## Conclusion

The use of different eGFR equations yielded clinically relevant differences in the estimation of GFR, potentially impacting drug dose adjustments. Comparisons between the CG equation and other equations demonstrated higher discrepancies. However, when comparing patient-adjusted and BSA-normalized versions of the equations, the discrepancies were not significant, particularly among patients with BSA close to the average. Apparently, CKD-EPI presented the best performance for drug dose adjustments in primary care.

### Supplementary Information

Below is the link to the electronic supplementary material.

Supplementary file1 (PDF 401 kb)

Supplementary file2 (PDF 1369 kb)
