# Supplementary material for: Discrepancies among equations to estimate the glomerular filtration rate for drug dosing decision making in aged patients: a cross sectional study
Source: Int J Clin Pharm. 2023 Dec 27;46(2):411–20. doi: 10.1007/s11096-023-01677-8 (PMC10960755; doi:10.1007/s11096-023-01677-8)
Supplement: Supplementary file 1 — Supplementary file1 (PDF 401 kb) [file 11096_2023_1677_MOESM1_ESM.pdf]

Castel-Branco MM, Lavrador M, Cabral AC, Pinheiro A, Fernandes J, Figueiredo IV, Fernandez-Llimos F. Discrepancies among equations to estimate the glomerular filtration rate for drug dosing decision making in aged patients.

Supplementary material S1. Bland-Altman plots analyzing the agreement between patient adjusted and body surface area-normalized equations to estimate glomerular filtration rate.

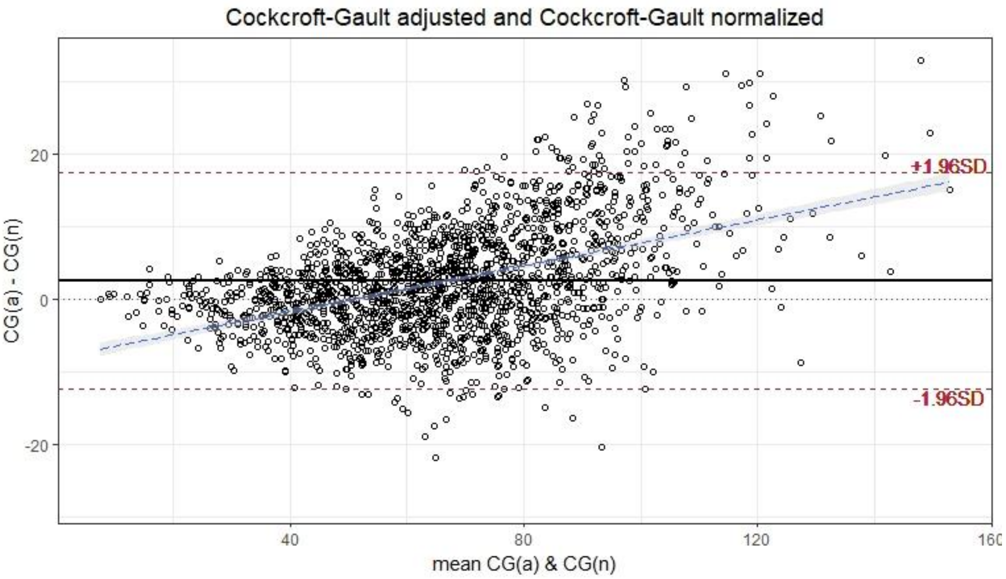

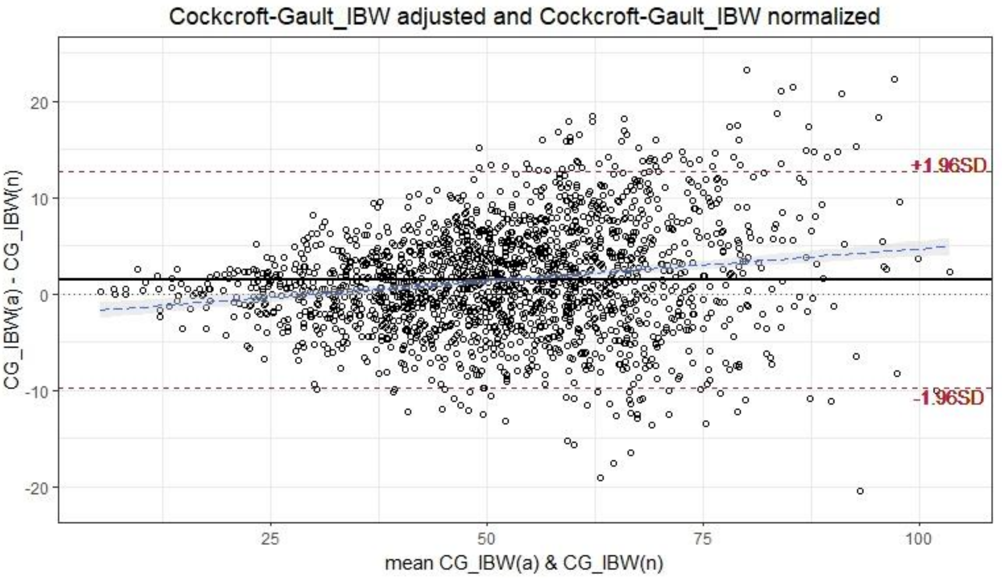

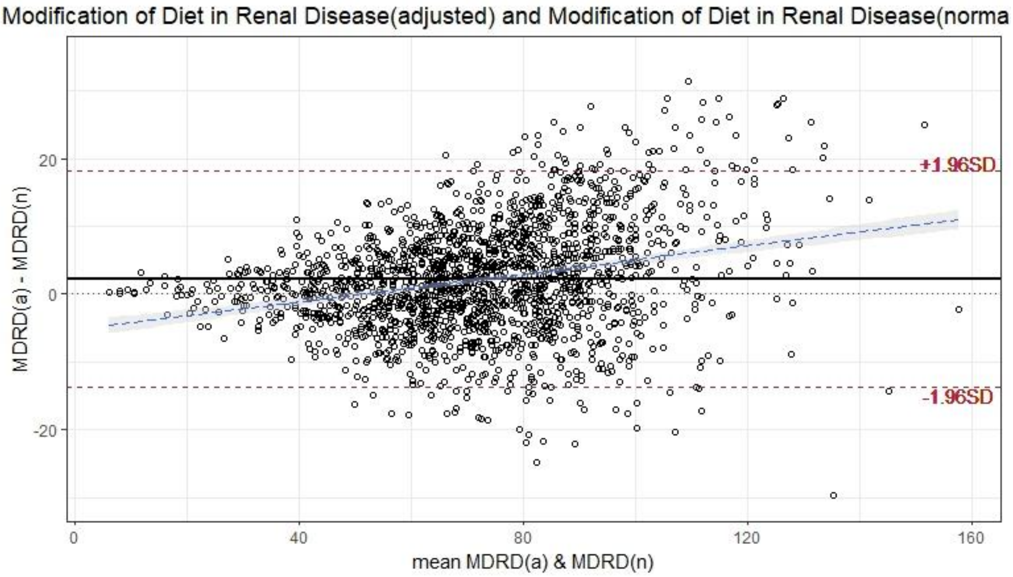

Modification of Diet in Renal Disease(adjusted) and Modification of Diet in Renal Disease(norma
MDRD(a) - MDRD(n)
mean MDRD(a) & MDRD(n)
+1.96SD
-1.96SD
20
0
-20
0
40
80
120
160

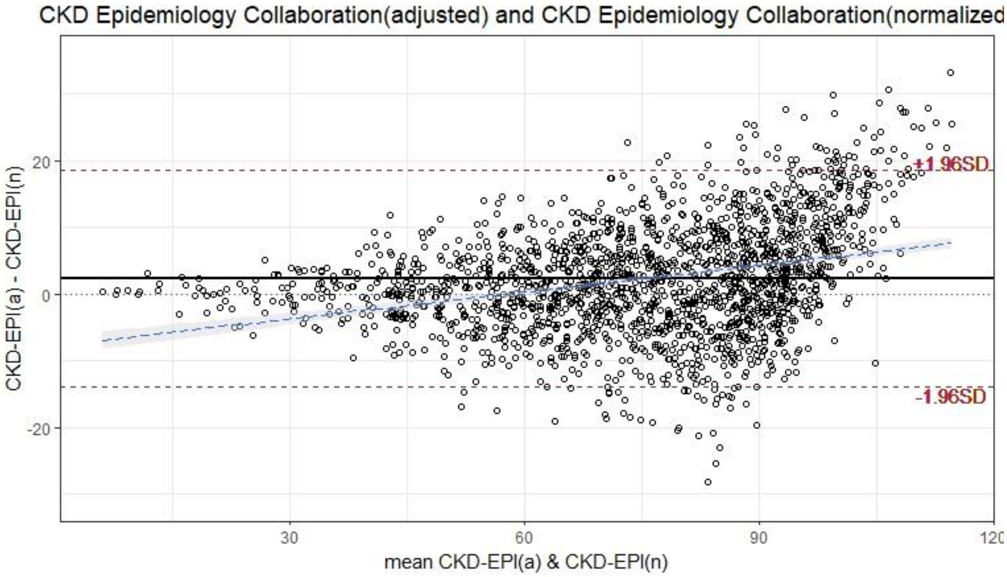

CKD Epidemiology Collaboration(adjusted) and CKD Epidemiology Collaboration(normalized
CKD-EPI(a) - CKD-EPI(n)
mean CKD-EPI(a) & CKD-EPI(n)
+1.96SD
-1.96SD
20
0
-20
30
60
90
120

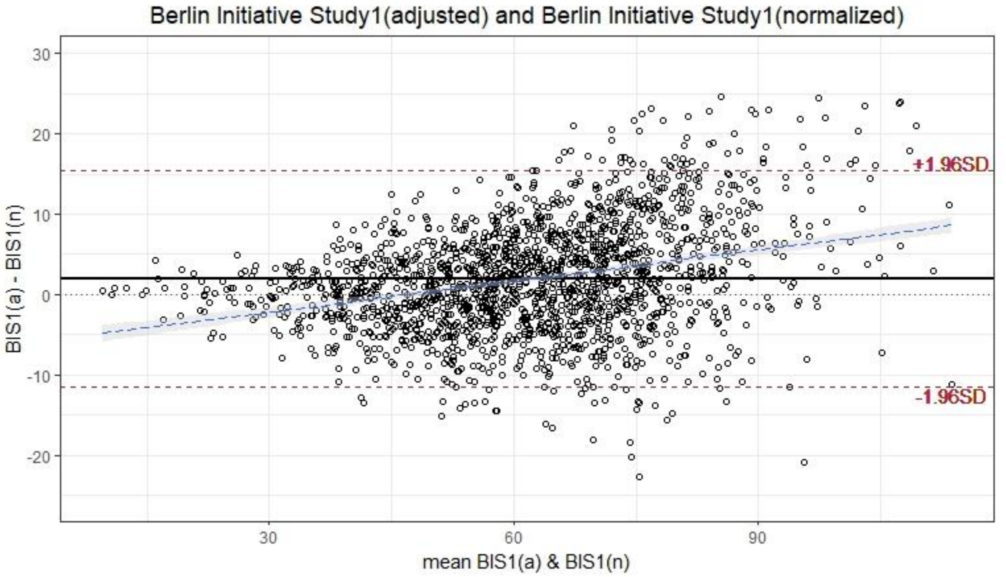

Berlin Initiative Study1(adjusted) and Berlin Initiative Study1(normalized)
BIS1(a) - BIS1(n)
mean BIS1(a) & BIS1(n)
+1.96SD
-1.96SD
30
20
10
0
-10
-20
30
60
90
