# Supplementary material for: Discrepancies among equations to estimate the glomerular filtration rate for drug dosing decision making in aged patients: a cross sectional study
Source: Int J Clin Pharm. 2023 Dec 27;46(2):411–20. doi: 10.1007/s11096-023-01677-8 (PMC10960755; doi:10.1007/s11096-023-01677-8)

Castel-Branco MM, Lavrador M, Cabral AC, Pinheiro A, Fernandes J, Figueiredo IV, Fernandez-Llimos F. Discrepancies among equations to estimate the glomerular filtration rate for drug dosing decision making in aged patients.

Supplementary File S2. Bland-Altman plots analyzing the agreement between original equations to estimate glomerular filtration rate.

			Limit of agreement		Regression		X when y=0	
Pairwise comparison	Bias	Error (%)	upper	lower	R Square	equation	X	95%CI
CG(adjusted) vs. CG_IBW(adjusted)	14.9	69.44	38.51	-8.66	0.386	y=0.38x -8.03	21	[17 : 26]
CG(adjusted) vs. MDRD(normalized)	-3.7	90.30	26.97	-34.37	0.386	y=0.17x -15.88	91	[65 : 130]
CG(adjusted) vs. CKD-EPI(normalized)	-6.0	84.86	22.85	-34.80	0.142	y=0.28x -25.49	93	[76 : 114]
CG(adjusted) vs. BIS1(normalized)	6.7	79.64	33.76	-20.34	0.401	y=0.46x -23.11	50	[44 : 57]
CG_IBW(adjusted) and CG(adjusted)	-14.9	89.00	8.66	-38.51	0.386	y=-0.38x +8.03	21	[17 : 26]
CG_IBW(adjusted) vs. MDRD(normalized)	-18.6	71.68	0.38	-37.62	0.167	y=-0.22x -4.93	-22	[-26 : -18]
CG_IBW(adjusted) vs. CKD-EPI(normalized)	-20.9	59.28	-5.18	-36.60	0.066	y=-0.12x -13.28	-111	[-120 : -104]
CG_IBW(adjusted) vs. BIS1(normalized)	-8.2	40.01	2.40	-18.81	0.045	y=0.07x -12.35	170	[130 : 231]
MDRD(normalized) and CG(adjusted)	3.7	85.61	34.37	-26.97	0.035	y=-0.16x +15.40	99	[66 : 153]
MDRD(normalized) and CG_IBW(adjusted)	18.6	53.02	37.62	-0.38	0.154	y=0.22x +4.93	-22	[-26 : -18]
MDRD(normalized) and CKD-EPI(normalized)	-2.3	34.73	10.16	-14.72	0.083	y=0.09x -9.17	97	[74 : 127]
MDRD(normalized) and BIS1(normalized)	10.4	40.90	25.08	-4.23	0.452	y=0.28x -8.38	30	[25 : 35]
CKD-EPI(normalized) and CG(adjusted)	6.0	77.97	34.80	-22.85	0.093	y=-0.25x +24.66	98	[77 : 126]
CKD-EPI(normalized) and CG_IBW(adjusted)	20.9	42.50	36.60	5.18	0.074	y=0.16x +11.25	-69	[-70 : -69]
CKD-EPI(normalized) and MDRD(normalized)	2.3	33.66	14.72	-10.16	0.204	y=-0.09x +9.17	97	[74 : 127]
CKD-EPI(normalized) and BIS1(normalized)	12.7	30.69	24.04	1.36	0.304	y=0.19x -0.06	0	[-4 : 5]
BIS1(normalized) and CG(adjusted)	-6.7	88.35	20.34	-33.76	0.302	y=-0.46x +23.57	52	[44 : 60]
BIS1(normalized) and CG_IBW(adjusted)	8.2	34.64	18.81	-2.40	0.007	y=-0.05x +11.53	254	[146 : 610]
BIS1(normalized) and MDRD(normalized)	-10.4	47.86	4.23	-25.08	0.007	y=-0.28x +8.38	30	[25 : 35]
BIS1(normalized) and CKD-EPI(normalized)	-12.7	37.05	-1.36	-24.04	0.304	y=-0.19x +0.06	0	[-4 : 5]

CG: Cockcroft-Gault; CG-IBW: Cockcroft-Gault with ideal body weight; MDRD: Modification of Diet in Renal Disease; CKD-EPI: Chronic Kidney Disease Epidemiology Collaboration; BIS1: Berlin Initiative Study 1; BSA: body surface area.

Castel-Branco MM, Lavrador M, Cabral AC, Pinheiro A, Fernandes J, Figueiredo IV, Fernandez-Llimos F. Discrepancies among equations to estimate the glomerular filtration rate for drug dosing decision making in aged patients.

Supplementary File S2. Bland-Altman plots analyzing the agreement between original equations to estimate glomerular filtration rate.

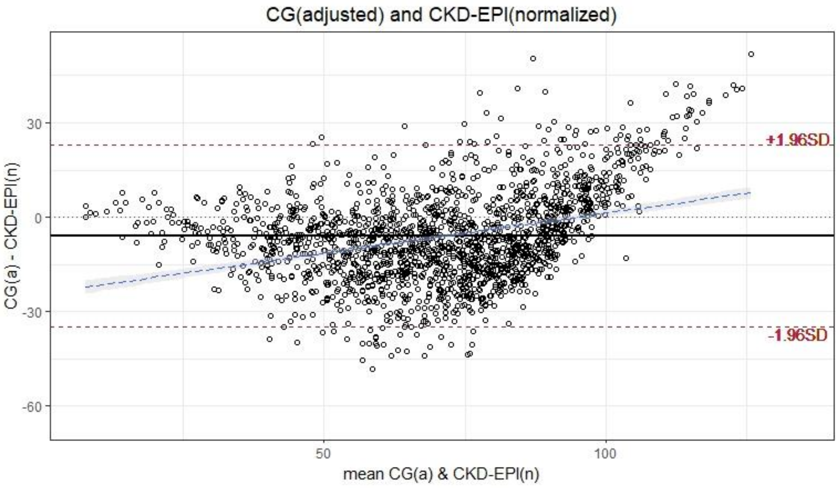

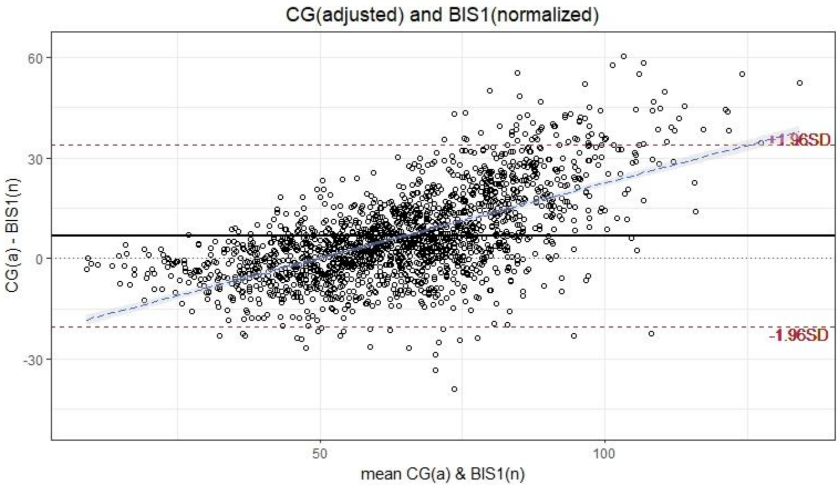

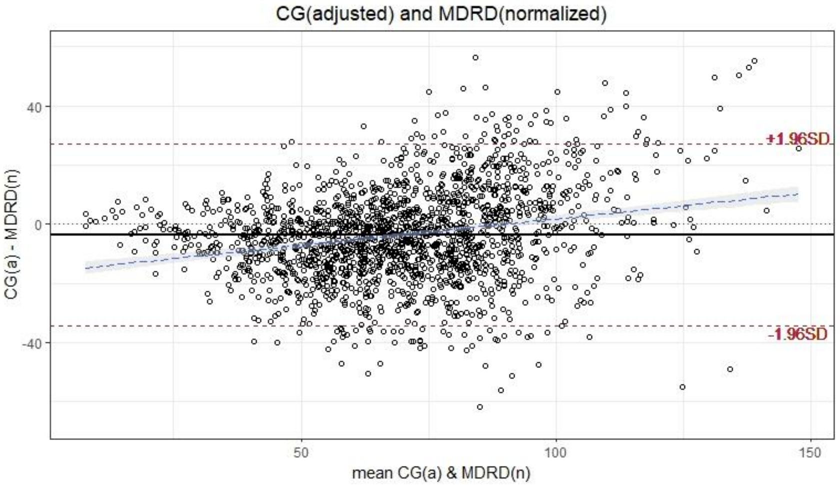

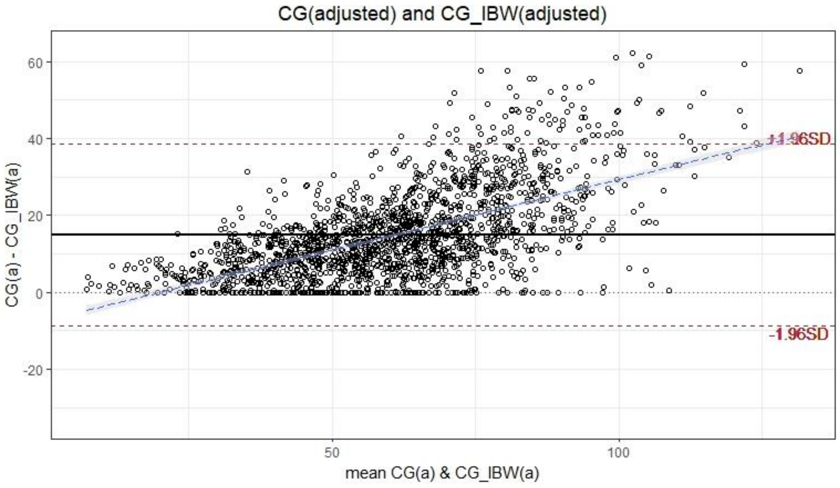

Castel-Branco MM, Lavrador M, Cabral AC, Pinheiro A, Fernandes J, Figueiredo IV, Fernandez-Llimos F. Discrepancies among equations to estimate the glomerular filtration rate for drug dosing decision making in aged patients.

Supplementary File S2. Bland-Altman plots analyzing the agreement between original equations to estimate glomerular filtration rate.

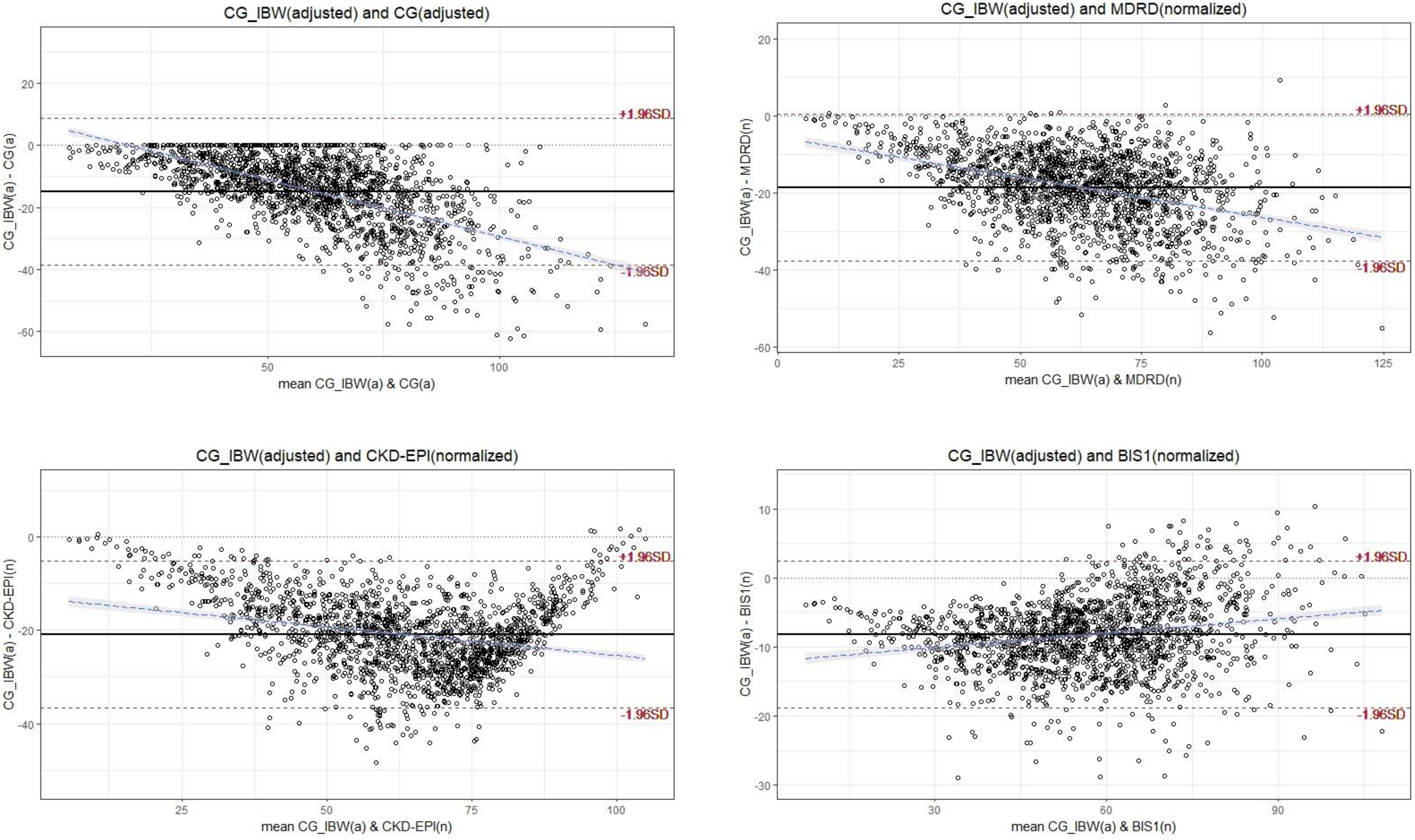

Castel-Branco MM, Lavrador M, Cabral AC, Pinheiro A, Fernandes J, Figueiredo IV, Fernandez-Llimos F. Discrepancies among equations to estimate the glomerular filtration rate for drug dosing decision making in aged patients.

Supplementary File S2. Bland-Altman plots analyzing the agreement between original equations to estimate glomerular filtration rate.

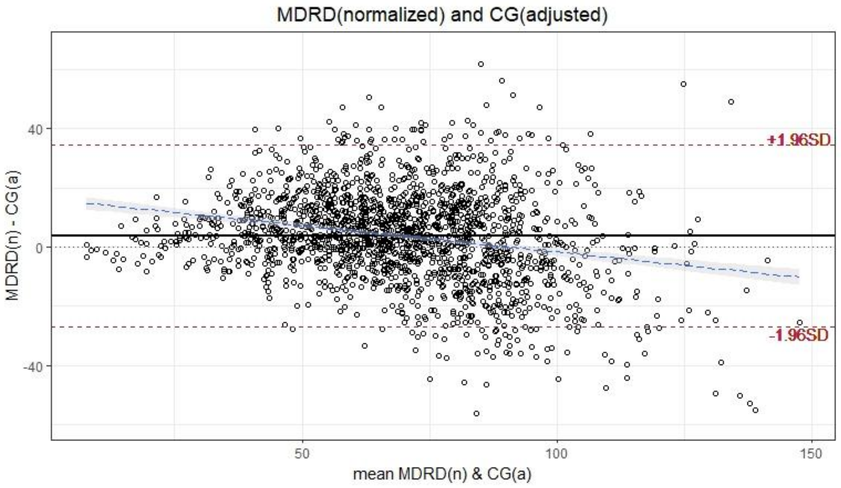

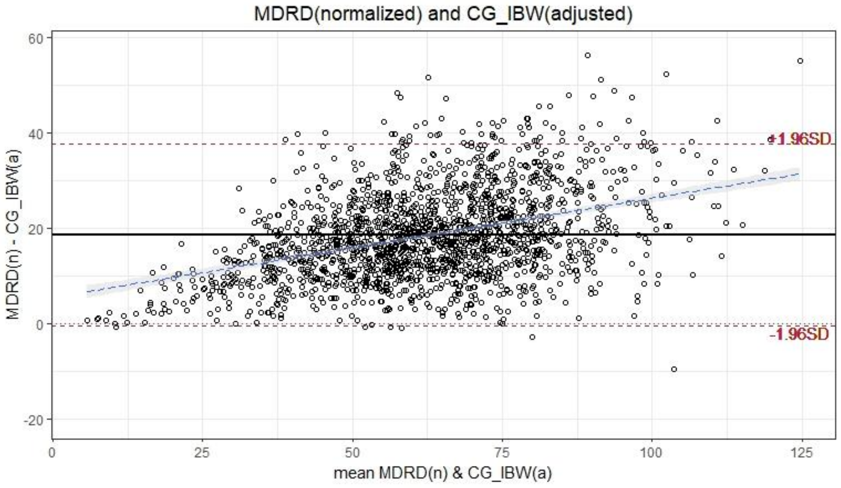

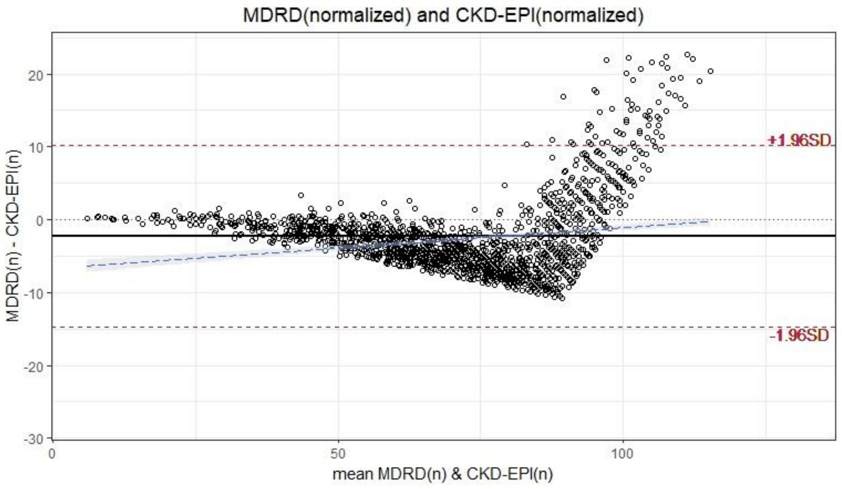

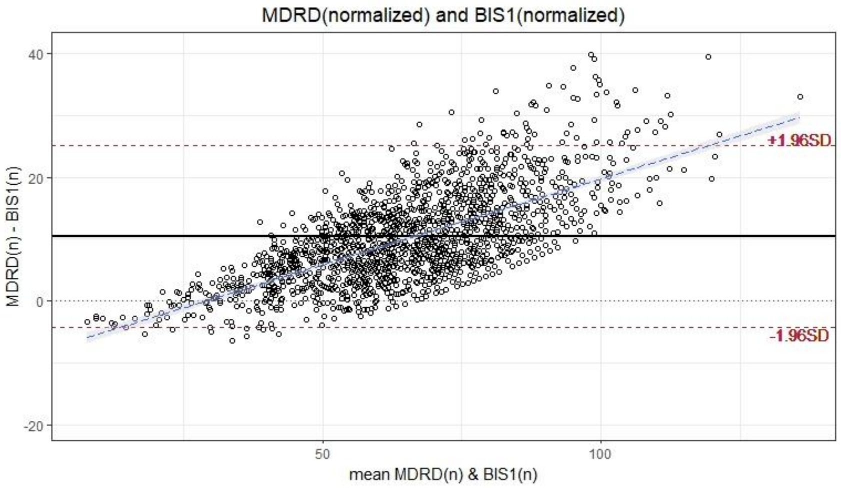

Castel-Branco MM, Lavrador M, Cabral AC, Pinheiro A, Fernandes J, Figueiredo IV, Fernandez-Llimos F. Discrepancies among equations to estimate the glomerular filtration rate for drug dosing decision making in aged patients.

Supplementary File S2. Bland-Altman plots analyzing the agreement between original equations to estimate glomerular filtration rate.

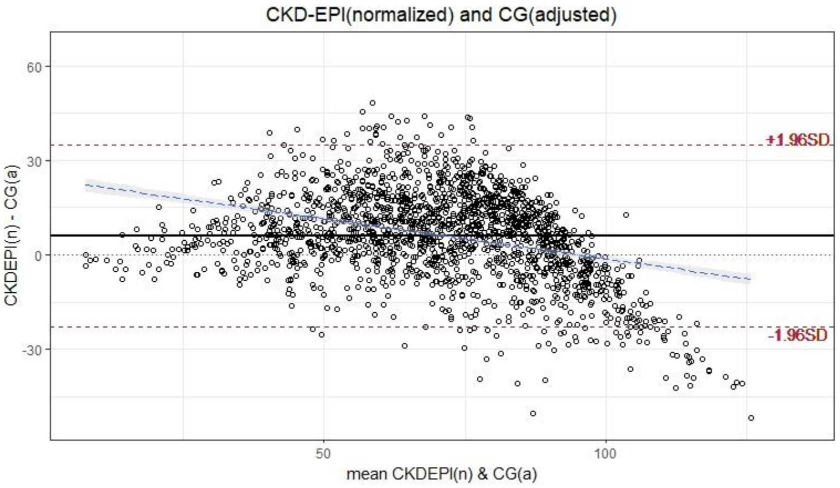

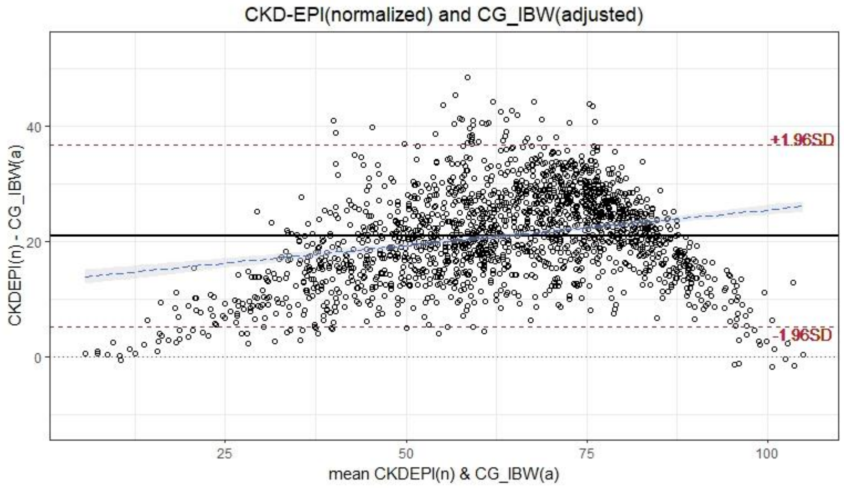

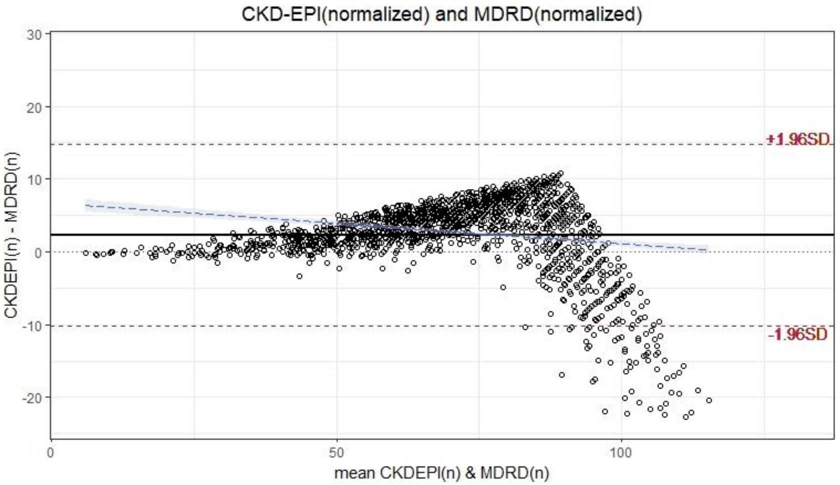

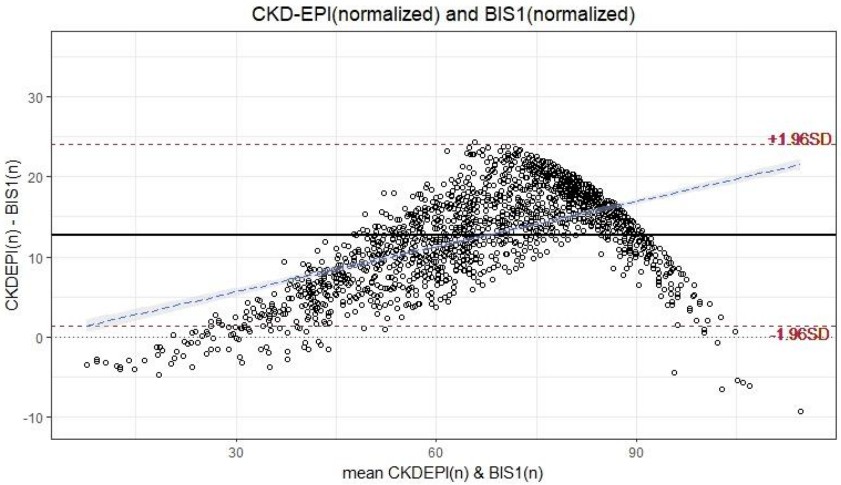

Castel-Branco MM, Lavrador M, Cabral AC, Pinheiro A, Fernandes J, Figueiredo IV, Fernandez-Llimos F. Discrepancies among equations to estimate the glomerular filtration rate for drug dosing decision making in aged patients.

Supplementary File S2. Bland-Altman plots analyzing the agreement between original equations to estimate glomerular filtration rate.

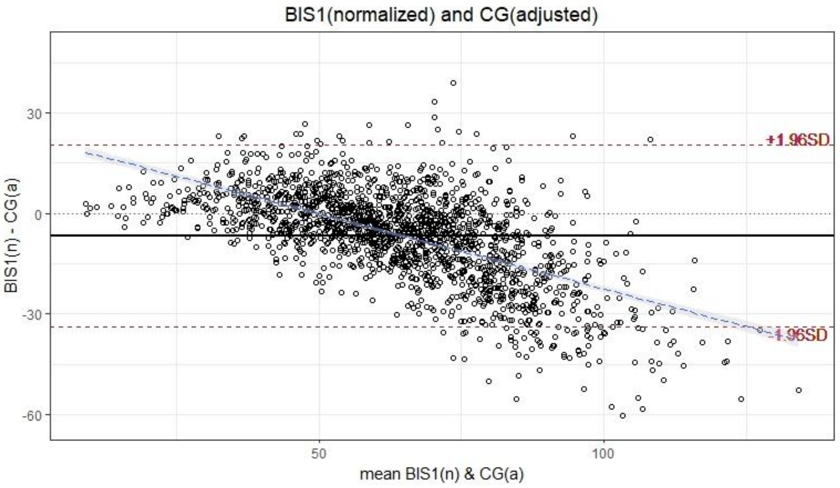

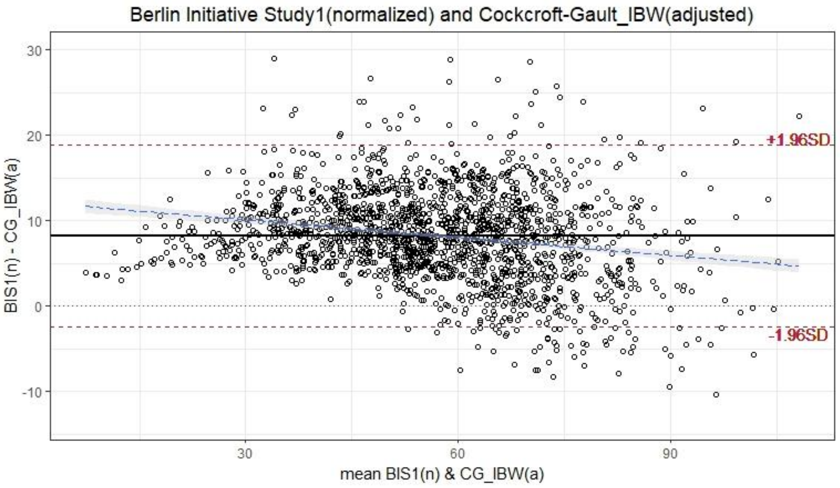

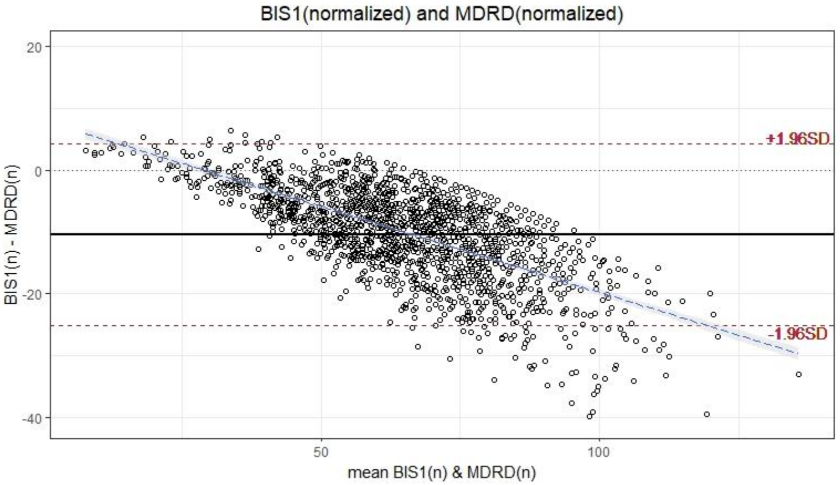

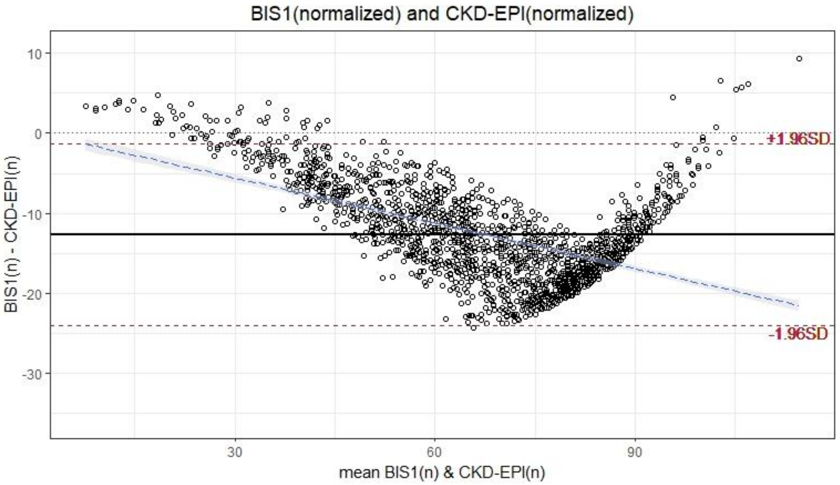

Supplement: Supplementary file 2 — Supplementary file2 (PDF 1369 kb) [file 11096_2023_1677_MOESM2_ESM.pdf]
